# ERTNet: an interpretable transformer-based framework for EEG emotion recognition

**DOI:** 10.3389/fnins.2024.1320645

**Published:** 2024-01-17

**Authors:** Ruixiang Liu, Yihu Chao, Xuerui Ma, Xianzheng Sha, Limin Sun, Shuo Li, Shijie Chang

**Affiliations:** ^1^School of Intelligent Medicine, China Medical University, Shenyang, Liaoning, China; ^2^Shanghai Institute of Microsystem and Information Technology, Chinese Academy of Sciences, Shanghai, China; ^3^School of Life Sciences, China Medical University, Shenyang, Liaoning, China

**Keywords:** EEG, emotion recognition, deep learning, transformer, interpretability

## Abstract

**Background:**

Emotion recognition using EEG signals enables clinicians to assess patients’ emotional states with precision and immediacy. However, the complexity of EEG signal data poses challenges for traditional recognition methods. Deep learning techniques effectively capture the nuanced emotional cues within these signals by leveraging extensive data. Nonetheless, most deep learning techniques lack interpretability while maintaining accuracy.

**Methods:**

We developed an interpretable end-to-end EEG emotion recognition framework rooted in the hybrid CNN and transformer architecture. Specifically, temporal convolution isolates salient information from EEG signals while filtering out potential high-frequency noise. Spatial convolution discerns the topological connections between channels. Subsequently, the transformer module processes the feature maps to integrate high-level spatiotemporal features, enabling the identification of the prevailing emotional state.

**Results:**

Experiments’ results demonstrated that our model excels in diverse emotion classification, achieving an accuracy of 74.23% ± 2.59% on the dimensional model (DEAP) and 67.17% ± 1.70% on the discrete model (SEED-V). These results surpass the performances of both CNN and LSTM-based counterparts. Through interpretive analysis, we ascertained that the beta and gamma bands in the EEG signals exert the most significant impact on emotion recognition performance. Notably, our model can independently tailor a Gaussian-like convolution kernel, effectively filtering high-frequency noise from the input EEG data.

**Discussion:**

Given its robust performance and interpretative capabilities, our proposed framework is a promising tool for EEG-driven emotion brain-computer interface.

## 1 Introduction

Emotions represent one of the higher cognitive functions of the brain that directly responds to the current mental state of the body (Tyng et al., 2017). Electroencephalography (EEG) emotion recognition aims to detect human emotional states by measuring and analyzing EEG signals. This approach has been broadly researched in the emotion recognition research field due to its high temporal resolution, non-invasiveness, and affordability (Gu et al., 2022; Tuncer et al., 2022). Regions of the brain, such as the orbitofrontal cortex, ventral media prefrontal cortex, amygdala, and hippocampal complex, have a close relationship with emotion generation and regulation, making EEG a dependable tool for emotion recognition (Phelps, 2004; Lindquist and Barrett, 2012). Previous studies have also demonstrated that EEG signals contain valuable information about current emotions, with the beta and gamma segments carrying the most significant information (Li et al., 2018; Pandey and Seeja, 2022). However, EEG signals also present challenges for feature extraction due to their instability, complexity, and limited spatial resolution.

Prior research, notably machine learning algorithms, has heavily relied on the quality of manually extracted features, which typically comprise signal statistical features (Jenke et al., 2014; Nawaz et al., 2020), power spectral density (PSD; Kollia, 2016), intrinsic mode functions (IMFs; Chen et al., 2019), spectrogram (Wang et al., 2019), wavelet coefficients (Aydin et al., 2016; Pandey and Seeja, 2022), fractal dimensional features (Tuncer et al., 2021), and entropy features (Li et al., 2022), among others.

Deep learning has emerged as a key methodology in the field of brain-computer interfaces due to its ability to bypass complex manual feature extraction and improve accuracy. In the task of EEG emotion recognition, convolutional neural networks (CNNs) are widely used due to their efficiency in extracting localized spatio-temporal features, EEGNet (Lawhern et al., 2018) is still considered one of the most reliable tool for EEG signal analysis. In addition, the EEG emotion recognition model based on improved CNNs by Liu et al. (2020), Zhu et al. (2021), Ding et al. (2022), and Chen et al. (2023) remains competitive. However, for EEG data characterized by long temporal dependencies, CNNs are obviously not the optimal choice. Techniques that feed EEG or processed features into LSTM networks, as demonstrated by Li et al. (2018), Xing et al. (2019), and Chakravarthi et al. (2022), yield superior results compared to CNNs. In addition, more advanced deep learning methods are being applied to EEG emotion recognition. These include the TC-Net network proposed by Wei et al. (2023). which combines Transformers and CapsNet and effectively captures both global and local features. In addition, the introduction of graph neural networks by Li et al. (2023) and Liu et al. (2023) to extract higher-order spatial features for EEG emotion recognition is a significant advance.

Beyond normal EEG emotion recognition, medical and clinical applications are attracting more attention (Pepa et al., 2023). Emotion recognition plays an important role in Cognitive Behavioral Therapy (CBT; Carpenter et al., 2018), Emotion Regulation Therapy (ERT)/Emotion-Focused Therapy (EFT; Lane et al., 2015), and the evaluation of medical treatment for emotion-related mental disorders, such as Generalized Anxiety Disorder (Goodwin et al., 2017) and Depression (Duman et al., 2016). EEG-based emotion brain-computer interfaces hold valuable as a tool for detecting affective feedback in remote healthcare (Tripathi et al., 2022). Huang et al. (2021) designed an emotion brain-computer interface system for patients with consciousness disorders, allowing them to express emotions through the interface. Wang et al. (2023) aim to understand the emotional cognition of hearing-impaired patients who are deprived of auditory information for long periods of time through EEG emotion recognition. However, the “black box” nature of deep learning poses a challenge to its further application. The Explainable AI (XAI) in EEG emotion recognition will be a critical area of future research. Not only will it help researchers validate existing medical knowledge or discover new ones, as Mayor Torres et al. (2023) using the explainable deep learning algorithm SincNet to identify high-alpha and beta suppression in EEG signals of individuals with autism spectrum disorders, but it will also increase physicians’ confidence in using deep learning for diagnosis (Jafari et al., 2023).

In this study, we proposed an interpretable end-to-end framework for EEG emotion recognition based on the transformer architecture. The framework uses EEG signals as input without the need for complex feature extraction and is both lightweight and interpretable. The remainder of the manuscript is organized as follows. Section 2.1 provides an introduction to the datasets used, including details on the employed preprocessing methods. In section 2.2, we introduce emotion recognition transformer network (ERTNet). Section 3 discusses the evaluations of our framework. Section 4 provides a comprehensive discussion, while section 5 details the conclusion.

## 2 Materials and methods

### 2.1 Datasets and preprocessing

Several institutions offer EEG datasets that can be used to train and validate emotion recognition models, such as DEAP (Koelstra et al., 2012), SEED (Wei-Long Zheng and Bao-Liang Lu, 2015), SEED-IV (Zheng et al., 2019), SEED-V (Liu et al., 2022), MAHNOB-HCI (Soleymani et al., 2012), and other datasets. These datasets elicit different emotional states by exposing participants to videos or audio stimuli. To assess the effectiveness of our proposed model, we conducted experiments using both the DEAP and SEED-V datasets. The diversity and richness of these datasets enhance our ability to evaluate the model’s generalization ability and robustness, providing a reliable foundation for future clinical applications.

#### 2.1.1 DEAP

**Figure 1:**
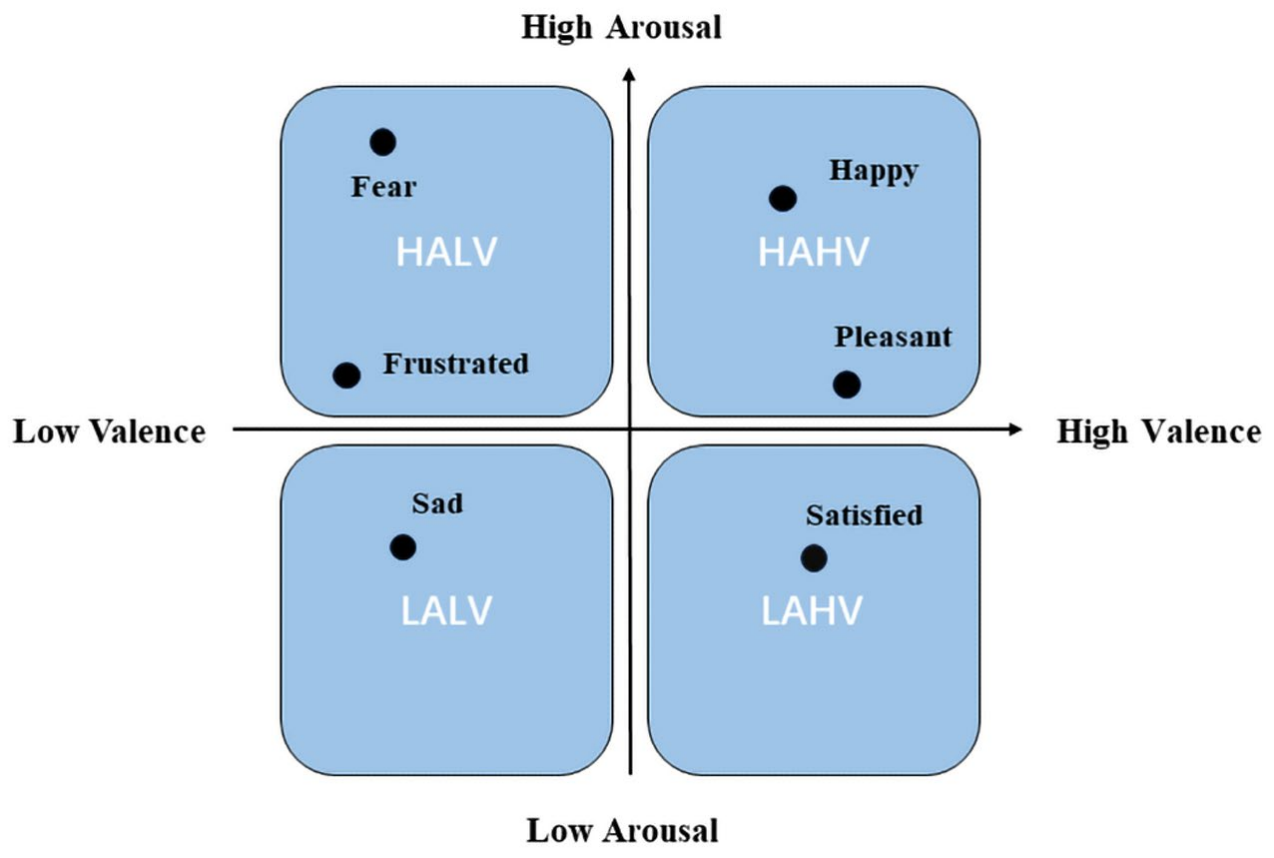
Arousal-valence space.

The DEAP dataset, collected by Queen Mary University of London, United Kingdom, is a multimodal dataset that documents 32 EEG leads and 8 other physiological signals (Koelstra et al., 2012). This dataset records physiological signals evoked by music videos. We utilized processed EEG data that had undergone a band-pass filter of 4 to 45 Hz, with removed EOG physiological artifacts, and resampled to 128 Hz. These EEG signals were segmented into non-repetitive 4-s segments for model evaluation. Emotion labels were assigned based on the Valence-Arousal-based approach (Russell, 1980) was chosen, as shown in Figure 1, classifying emotions into four categories: high arousal, high valence (HAHV), low arousal, high valence (LAHV), high arousal, low valence (HALV), and low arousal, low valence (LALV).

#### 2.1.2 SEED-V

The SEED-V dataset, provided by the Laboratory of Brain-like Computing and Machine Intelligence at Shanghai Jiao Tong University, comprises emotional states categorized into five categories, collected from 16 subjects of a 1:1 male-to-female ratio. The subjects’ EEG signals were recorded while they watched movie clips, and the dataset includes emotions such as “happy,” “sad,” “disgust,” “fearful,” and “neutral.” EEG signals were recorded in a total of 62 channels in accordance with the international 10–20 system (Liu et al., 2019).

The preprocessing was performed using the same steps as outlined in Liu et al. (2022) and Wu et al. (2020), we applied several preprocessing steps to the data, including (1) removal of 50 Hz industrial frequency interference using MNE-Python (Gramfort, 2013); (2) elimination of physiological artifacts using the EOG and FPZ channels provided in the dataset; (3) application of the FIR bandpass filter provided by MNE-Python to remove signals outside the 1–50 Hz range; (4) segmentation of the data into non-repeating 4-s segments; and (5) resampling of the signal to 200 Hz and normalization.

### 2.2 Framework

**Figure 2:**
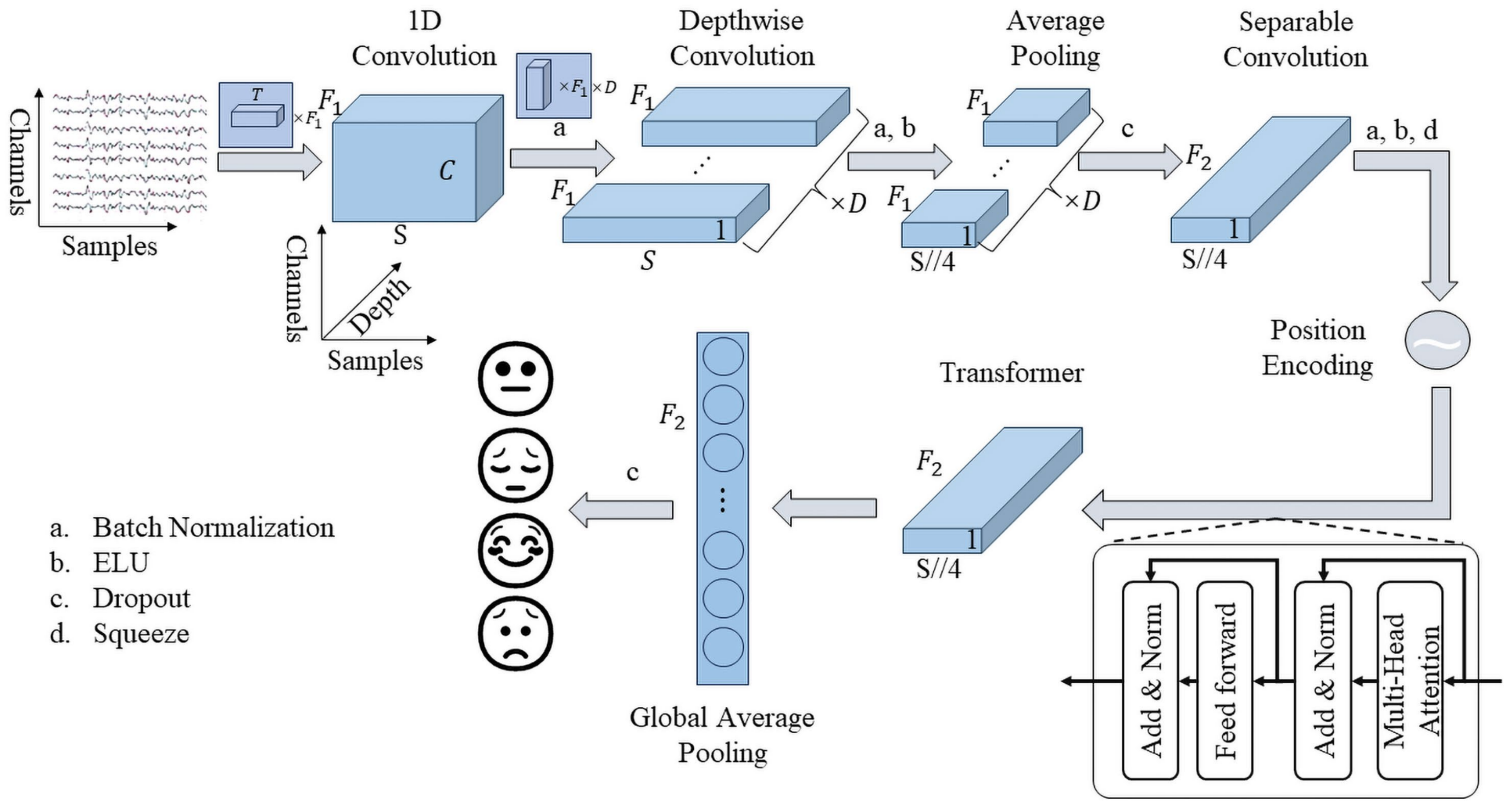
Framework of ERTNet.

Figure 2 depicts the general structure of our proposed framework, named ERNet. Its core components consist of a feature extraction module and a transformer module.

The feature extraction module, similar to EEGNet, predominantly utilizes spatio-temporal convolution techniques. The initial layer employs 1D convolution to remove noise and extract features. A batch normalization layer is inserted between the first and second layer to address the issue of vanishing gradients. However, to maintain the interpretability of the spatiotemporal convolution, an activation function is excluded from the first convolutional layer. The second layer comprises of a spatial convolution layer, which merges the valid information on all channels. The spatial convolution is separated in depth dimension, achieved through depthwise convolution in the code. The activation function is recovered while adding the batch normalization layer between the second and third layer. The third layer comprises an average pooling layer, which pools with a length of 4. In order to avoid overfitting, a dropout layer is included after the third layer. Following both batch normalization and dropout layer, the last layer, a high-level feature fusion layer is employed. This layer leverages a separable convolutional layer implementation, minimizing the number of parameters. Finally, the feature extraction module output will reduce in dimension to fit the transformer module.

Its feature extraction module has four important parameters, $F_{1}$ , T , D , and $F_{2}$ , denoting the number of temporal convolutional kernels, the length of temporal convolutional kernels, the number of spatial convolutional kernels, and the number of pointwise convolutional kernels in the separable convolutional layer, respectively. Following EEGNet, we set $F_{2}=F_{1}\times D$ .

The transformer module is derived from the encoder section of the transformer structure proposed by Google in 2017 (Vaswani et al., 2017). It employs position encoding at the connection between the transformer block and the feature extraction part to help parallel multi-head attention to focus on temporal information. The transformer module comprises a multi-head attention layer and a feed-forward layer, incorporating a residual structure and layer normalization layer present at their output. To minimize the number of parameters, the feed-forward layer uses a single neural network layer. The multi-head attention layer is the core component of the transformer module to help the model better integrate the features of the output of the feature extraction part. For an input $X\in R^{d\times F_{2}}$ , the output of the multi-head attention is computed as:


$$\mathrm{MutiHeadAttention}\left(Q,\;K,\;V\right)=\mathrm{Concat}\left(head_{1},\;\cdots ,\;head_{n}\right)W^{O}$$

$$head_{i}=\mathrm{softmax}\left(\frac{\left(XW_{i}^{Q}\right){\left(XW_{i}^{K}\right)}^{T}}{\sqrt{d_{k}}}\right)\left(XW_{i}^{V}\right)$$

where n denotes the number of self-attentive heads, n=8 in our experiments, $\mathrm{Concat}\left(\cdot \right)$ denotes the merge operation, and $W_{i}^{Q}$ , $W_{i}^{K}$ , $W_{i}^{V}\in R^{F_{2}\times F_{2}}$ , $W^{O}\in R^{nF_{2}\times F_{2}}$ . The output of the transformer module incorporates a global average pooling layer in place of the flatten layer to decrease the number of parameters, and it is then connected to the decision layer.

Moreover, the dropout rate is set to 0.5 in subject-dependent experiment but 0.25 in subject-independent experiment, following previous research suggestions, and the activation function of all layers is unified with the “ELU” function, defined as:


$$ELU\left(x\right)=\{\begin{array}{c} x,x>0\\ \alpha \left(e^{x}-1\right),x\leq 0 \end{array}$$

where α represents decay factor when x is negative.

## 3 Results

### 3.1 Experiment setups

Our model was trained using a NVIDIA Quadro RTX 8000 GPU and implemented in Python using the Tensorflow-Keras library. In the DEAP dataset, due to the limited amount of data, all data were used for 10-fold cross-validation. However, the SEED-V dataset is partitioned into train and test sets in an 8:2 ratio, with results analyzed using the Wilcoxon-test for the 10-fold cross-validation on the train set and the Delong-test for the test set. Prior to model training, we utilized the hyperparameter optimization method offered by the Optuna library (Akiba et al., 2019) to explore F1 , T , D , and attention heads within the ranges of [4, 20], [5, 100], [1, 5], and [4, 20], respectively. To enhance the evaluation of ERTNet’s performance, we conducted subject-independent experiments that build upon the previous subject-dependent experiments. We retained identical hyperparameters and data processing, and implemented Leave-One-Subject-Out Cross Validation (LOSOCV), as previously suggested (Ding et al., 2022).

### 3.2 Model evaluation

#### 3.2.1 Experiment 1

**Figure 3:**
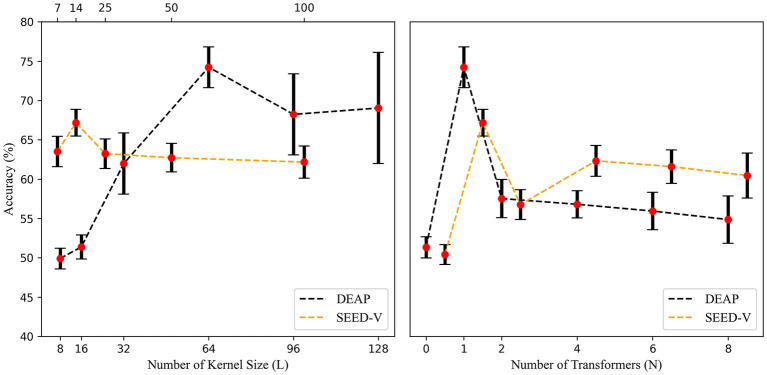
Accuracy with different temporal kernel lengths and number of transformer blocks. To prevent disturbances in the image display, we shifted the curve of SEED-V 0.5 units to the right in the right side of the figure.

In this section, we conducted a preliminary screening for the model through the hyperparameter optimization method provided by Optuna. In the DEAP dataset, the final optimal hyperparameters were $F_{1}=8$ , T=64 , D=4 , $F_{2}=32$ , while for SEED-V, they were $F_{1}=16$ , T=14 , D=4 , $F_{1}=64$ . As the results of the hyperparameter optimization of T were different on the two datasets, we repeated the test on the DEAP and SEED-V dataset, confirming the suitability of a large convolutional kernel for DEAP and a small for SEED-V. Figure 3 shows that the two datasets require dissimilar lengths of convolutional kernels to attain higher accuracy. Furthermore, Figure 3 demonstrates the impact of different numbers of Transformer Blocks, revealing that a single transformer block fulfills the requirement, and additional blocks diminish accuracy. In the next section, we use EEGNet ( $F_{1}$ ( T ), D ) denotes the hyperparameters in EEGNet, while the feature extraction part of our proposed model shares the same hyperparameters.

#### 3.2.2 Experiment 2

##### 3.2.2.1 Subject-dependent

**Figure 4:**
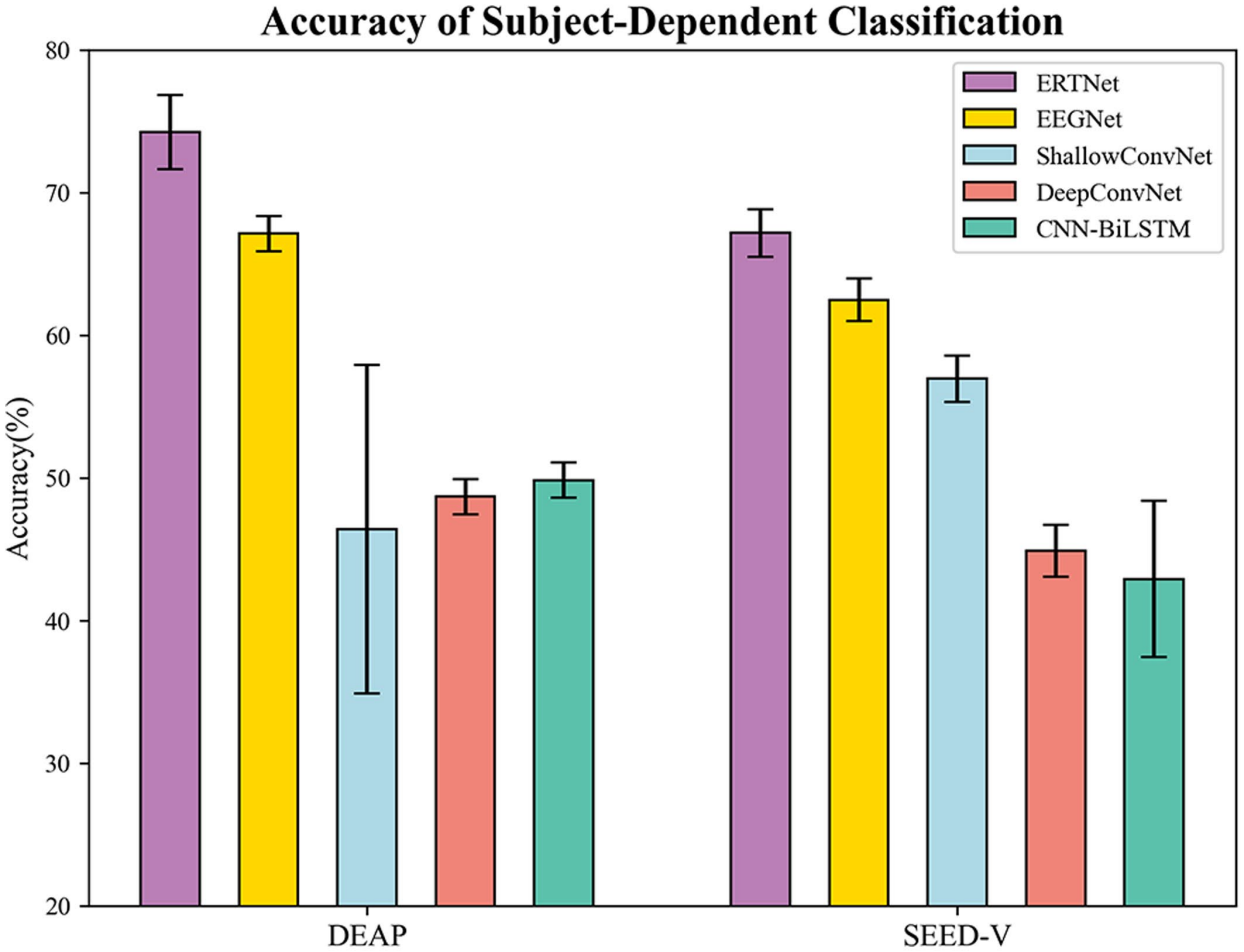
Accuracy of subject-dependent on DEAP and SEED-V.

We compared our model with models based on convolutional neural networks, including EEGNet (Lawhern et al., 2018), DeepConvNet, and ShallowConvNet (Schirrmeister et al., 2017), as well as the model based on recurrent neural networks CNN-BiLSTM (Zhang et al., 2020). The results of the 10-fold cross-validation are shown in Figure 4. On the DEAP dataset, our proposed transformer-based model achieved an accuracy of 74.23% ( ± 2.59%), significantly outperforming the other baselines. Moreover, the average AUC value of the model is 0.93, indicating the model’s excellent performance on the emotion classification task. Conversely, the average accuracy of EEGNet (8 (64), 4), CNN-BiLSTM, ShallowConvNet, and DeepConvNet were 67.12%, 49.83%, 46.40%, and 48.68%, respectively, all of which were lower than our model ( p<0.01 ).

**Figure 5:**
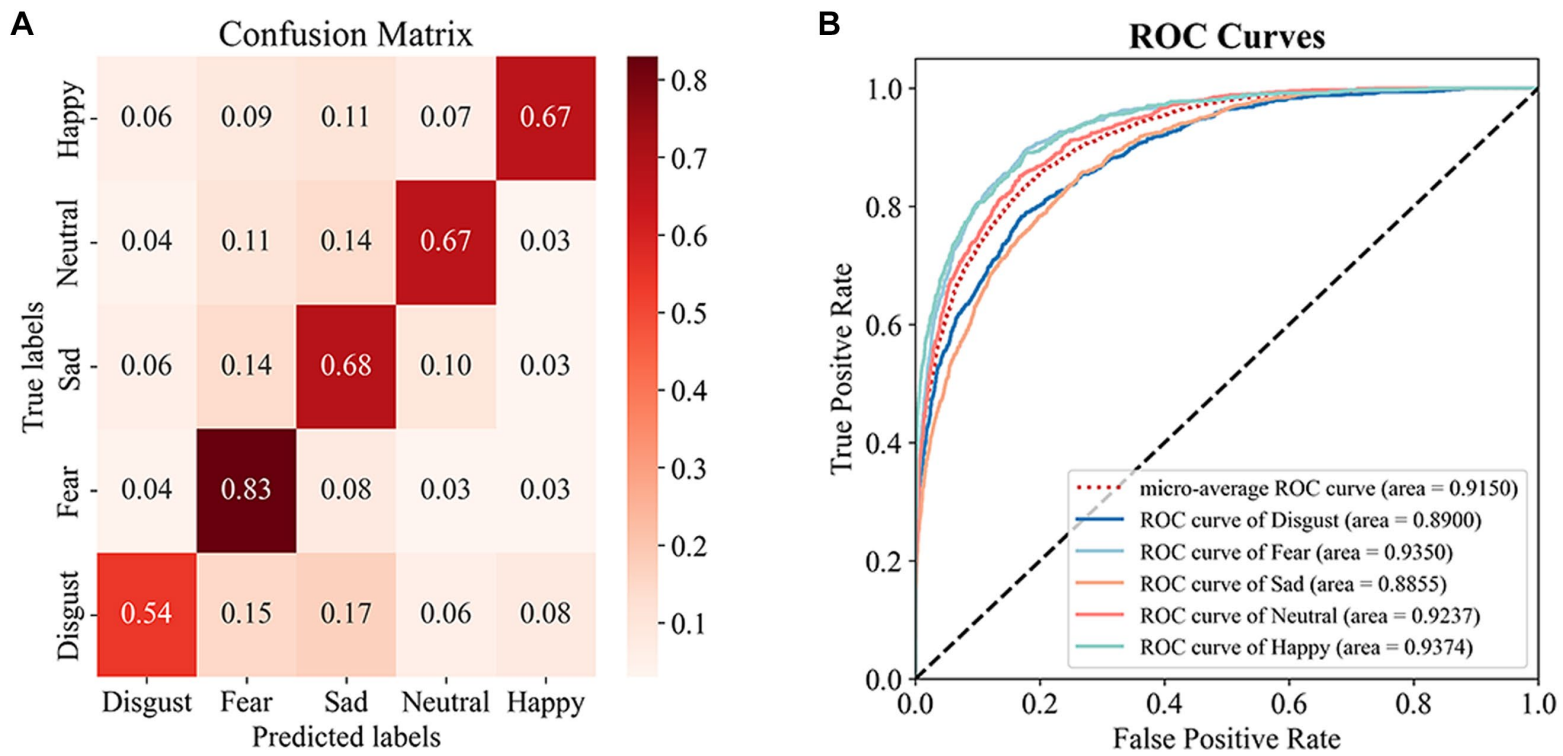
Results in SEED-V test set. **(A)** Confusion Matrix, **(B)** ROC Curves.

We utilized a 10-fold cross-validation technique to assess the model’s performance on the SEED-V dataset. To expedite the model’s convergence, we applied transfer learning by integrating the parameters of a tuned EEGNet model’s front-end feature extraction section directly into our model for training. The transformer-based model achieved an average accuracy of 67.17% ( ± 1.70%) and an average AUC value of 0.91 on this dataset, outperforming the other four models. EEGNet (16 (14), 4), CNN-BiLSTM, ShallowConvNet, and DeepConvNet exhibited average accuracies of 62.84%, 42.91%, 56.95%, and 44.88%, respectively ( p<0.01 ). On the test set, the top-performing transformer-based model attained an accuracy of 68.19% along with an AUC value of 0.91. Figure 5 depicts the confusion matrix and ROC curves providing the exceptional execution of our framework for the task of recognizing emotions.

##### 3.2.2.2 Subject-independent

**Figure 6:**
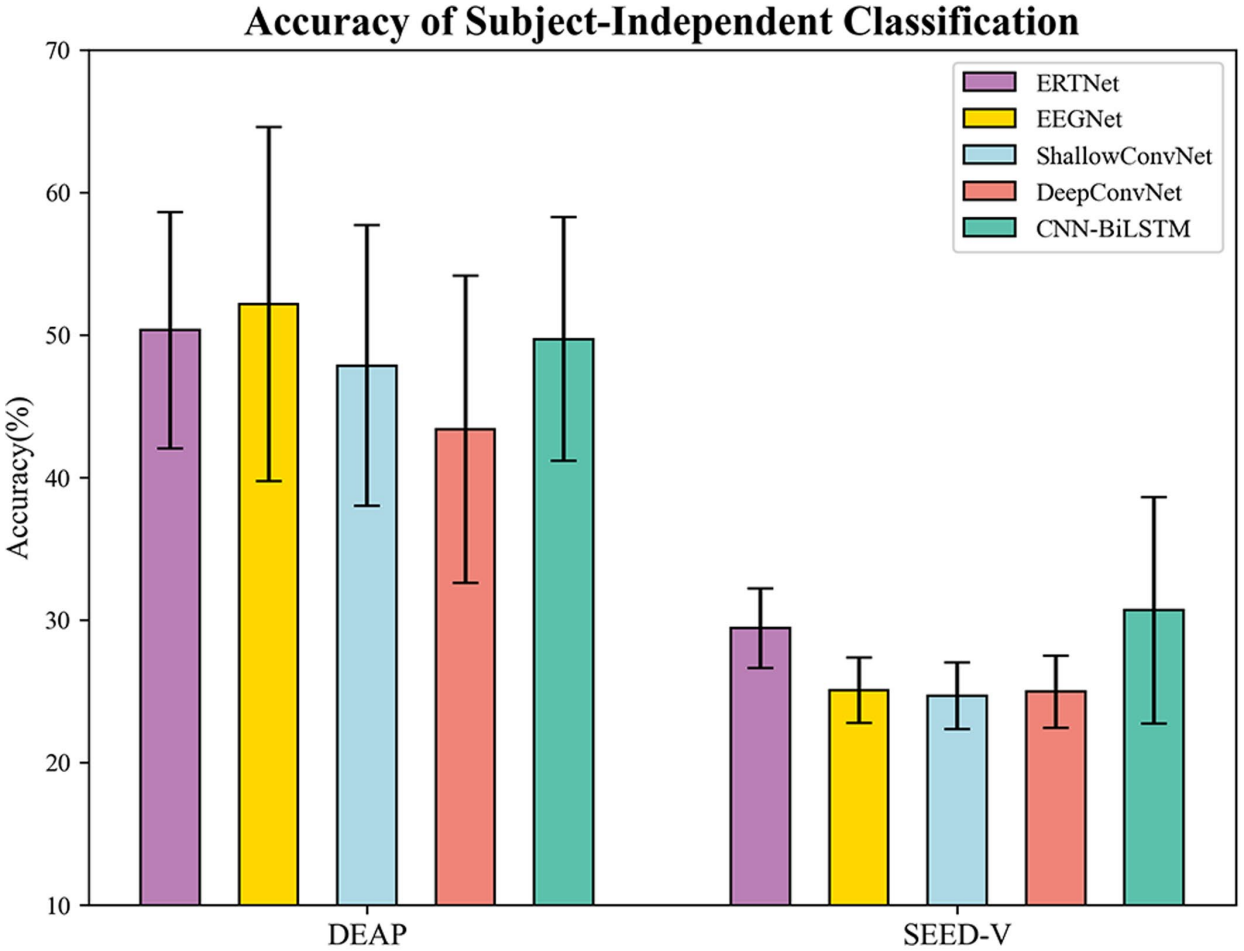
Accuracy of subject-independent on DEAP and SEED-V.

The results of subject-independent testing of the ERTNet model against four other models (EEGNet, ShallowConvNet, DeepConvNet, and CNN-BiLSTM) on both the DEAP and SEED-V datasets are presented in Figure 6. On the DEAP dataset, the average accuracies of ERTNet, EEGNet, ShallowConvNet, DeepConvNet, and CNN-BiLSTM are 50.33%, 52.14%, 47.84%, 43.37%, and 49.71%, respectively. On the SEED-V dataset, the average accuracies of these models are 29.41%, 25.07%, 24.66%, 24.96%, and 30.67%, respectively. The performance of ERTNet was evaluated and statistical analysis was performed using Wilcoxon tests to examine performance differences between models on each dataset.

The analysis of the DEAP dataset revealed significant performance differences between ERTNet and DeepConvNet ( p<0.01 ) and ShallowConvNet ( p<0.01 ). However, the differences with EEGNet (8 (64), 4; p=0.24 ) and CNN-BiLSTM ( p=0.18 ) were not significant. These results suggest that ERTNet outperforms DeepConvNet and ShallowConvNet on the DEAP dataset. The SEED-V dataset shows that ERTNet outperforms EEGNet (16 (14), 4), DeepConvNet, and ShallowConvNet ( p<0.01 ) in terms of performance, while the difference with CNN-BiLSTM ( p=0.50 ) is not statistically significant. These results confirm the effectiveness of ERTNet on various datasets, particularly when compared to EEGNet, DeepConvNet, and ShallowConvNet.

In subject-independent experiment, the ERTNet model demonstrated comparable or superior performance to several other models, although not to the same extent as in subject-dependent experiment. On the DEAP dataset, ERTNet exhibited significant performance gains compared to DeepConvNet and ShallowConvNet. On the SEED-V dataset, ERTNet demonstrated superior performance compared to the other models and was also comparable to CNN-BiLSTM. The findings indicate that ERTNet is an efficient neural network model for processing emotional information in EEG signals.

#### 3.2.3 Experiment 3

**Figure 7:**
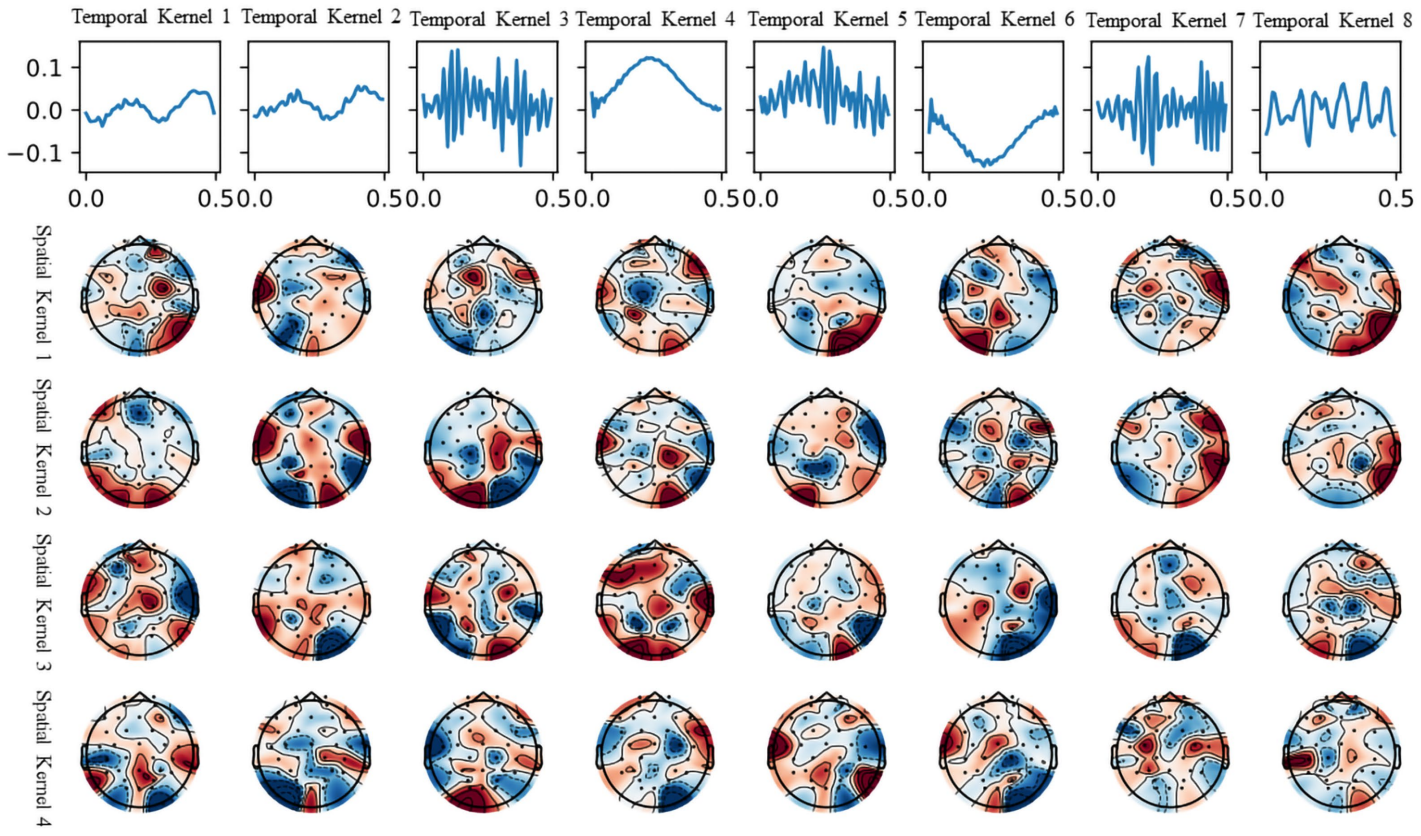
Visualization of temporal and spatial kernels from the trained model on DEAP.

In this section, we demonstrate the interpretability of the model trained on the DEAP dataset. We present a visualization of the convolutional kernels utilized in the feature extraction segment of the top-performing model from the 10-fold cross-validation procedure on the DEAP dataset. The visualization, displayed in Figure 7, aids in understanding the model’s concentration on different parts of the EEG signal during fitting.

The first row shows the eight time-domain convolution kernels of the model’s initial layer, covering all frequency ranges. It should be noted that not all convolutional kernels are utilized for beneficial information extraction. Temporal convolutional kernels 3, 5, and 7 are used to extract the high-frequency (beta and gamma) segments of the EEG signal, while convolutional kernel 8 is used to extract the mid-frequency (alpha) segment of the EEG signal. Temporal convolutional kernels 3, 5, and 7 are used to extract the high-frequency (beta and gamma) segments of the EEG signal, while convolutional kernel 8 is used to extract the mid-frequency (alpha) segment of the EEG signal. These convolutional kernels, working with their corresponding spatial convolution kernels in the brain terrain map, can divide the information into separate frequency bands and decipher the EEG signals on different channels.

The brain topography presented in the four rows corresponds to each of the four spatial convolution kernels within the second layer of the model. The spatial convolutional kernels for the high-frequency signal filters appear to be concentrated in the temporal and prefrontal regions, aligning with the understanding that these areas, including the amygdala and hippocampus, are crucial in emotion generation and recognition (Phelps, 2004). In addition, the production of emotions corresponds to higher cognitive activity in humans, with accompanying EEG signals primarily observed in the high-frequency (beta and gamma) bands, as evident in the temporal convolutional kernels. Similar findings have been supported by the study of Gong et al. (2023), which utilized visualization of attention masks.

Furthermore, we conducted an ablation study to evaluate the significance of each temporal convolutional kernel for classification using the same model trained on the DEAP dataset. Table 1 displays the findings, categorized into three sections. The initial section shows the outcomes after removing individual convolutional kernels one at a time. The second section depicts the results based on each convolutional kernel’s frequency domain similarity, while the third section showcases the results after not removing any convolutional kernel.

**Table 1:** Performance when temporal kernel(s) was/were removed, here all DEAP data is used.

Kernel(s) removed	AUC value				
	Average	HAHV	LAHV	HALV	LALV
1	0.9402	0.9244	0.9363	0.9508	0.9472
2	0.9334	0.9210	0.9334	0.9441	0.9266
3	0.8850	0.8970	0.9101	0.8959	0.9095
4	0.8193	0.8054	0.8094	0.8129	0.8129
5	0.8838	0.8861	0.8703	0.8923	0.9220
6	0.7971	0.7780	0.7962	0.8056	0.8053
7	0.8994	0.8848	0.9018	0.9173	0.8980
8	**0.9417**	**0.9338**	**0.9455**	**0.9496**	**0.9492**
1, 2	**0.9120**	**0.8943**	**0.9055**	**0.9268**	**0.9115**
4, 6	0.7084	0.6796	0.6837	0.7395	0.6803
3, 5, 7	0.7442	0.7197	0.7587	0.7507	0.7697
3, 5, 7, 8	0.7176	0.7127	0.7352	0.7241	0.7349
None	**0.9734**	**0.9635**	**0.9725**	**0.9760**	**0.9796**

Bold values indicate cases where kernel(s) is/are removed with minimal impact on the model.

**Figure 8:**
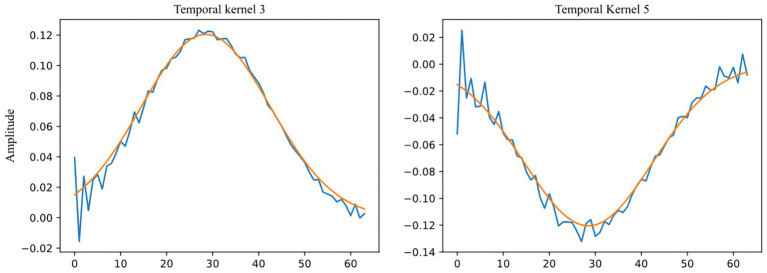
Comparison between Temporal Kernels 3, 5 (blue) and Gaussian kernel ( std.=14 , amplitude divided by 8.3, orange).

The ablation experiments indicate that removing a single temporal convolutional kernel has a relatively minor effect on the model when it comes to extracting information from EEG signals in the low and medium frequency bands, specifically from the three temporal convolutional kernels 1, 2, and 8. However, the removal of three temporal convolutional kernels—3, 5, and 7—that extract information from EEG signals in the high-frequency (beta and gamma) bands, has a considerable impact on the model. Interestingly, two Gaussian-like convolutional kernels, namely temporal kernels 4 and 6, have a greater impact on the model than even kernels 3, 5, and 7. We propose a hypothesis for this phenomenon: there may still be some high-frequency noises in the high-frequency band that have not been fully eliminated from the processed data provided by the DEAP dataset. During training, the model actively fits temporal convolutional kernels similar to the Gaussian kernel to provide a clearer and more stable signal input for the subsequent convolutional layers, thus enhancing the efficiency and accuracy of feature extraction. Supporting this hypothesis is the study conducted by Bertoni, which indicates the emergence of Gabor-like kernels (Gaussian kernels being a specialized type of Gabor kernel) in the shallow layers of convolutional neural networks during their examination of natural image research (Bertoni et al., 2021). This hypothesis further extends the findings of their research. Figure 8 shows the comparison of convolution kernels 3 and 5 with a Gaussian kernel with a standard deviation of 14 and a magnitude reduction of 8.3 times.

## 4 Discussion

The comparison of recognition accuracy using various convolutional kernel lengths on the DEAP dataset revealed that a kernel length of 64 provided the best performance. Given the intricate time-frequency details of EEG signals, the optimal kernel length for extracting vital frequency bands through convolution seems to be influenced by the input signal preprocessing technique and the emotion elicitation approach. The study determined that time-domain convolutional layer with larger kernels (length 64) performed superiorly in the DEAP dataset, whereas smaller kernels (length 14) were more appropriate for the SEED-V dataset. This difference in suitability may be linked to variations in preprocessing and emotion elicitation between the two datasets. Larger kernels can create improved Gaussian-like kernels, resulting in the removal of artifacts present in the DEAP dataset. SEED-V utilized a 1–50 Hz bandpass filter, which is wider than that used in DEAP’s preprocessing and has superior data quality. This makes it more appropriate for small kernels that concentrate on high-frequency beta and gamma bands. The connection between kernel length and the minimum detectable frequency is determined by the formula:


$$F_{\text{min}}=Sr/T$$

where $F_{\text{min}}$ means the minimum detectable frequency of the model, Sr means the EEG sampling rate, and T means the length of the temporal convolutional kernel. Tuning the length of the kernel to match the primary frequency bands related to emotion in the input signal can enhance the removal of artifacts and direct the model toward learning time-frequency patterns relevant to emotion.

Our proposed model outperforms CNN and LSTM models. Tables 2, 3 also presents the binary classification performance of our model for the valence and arousal dimensions, using identical hyperparameters for both models. The remaining data in the table showcases the performance of previous approaches on DEAP. The inclusion of the Transformer module makes possible the full consideration of local temporal and spatial features obtained by the feature extraction module through its multi-head attention mechanism. Current studies on SEED-V primarily focus on multimodal emotion recognition, making it impractical to make a comparable comparison. Still, this research could provide valuable insights into EEG signal processing within multimodal signal processing. The feature extraction component of our model has the potential to be applied to sub-models within multimodal models.

**Table 2:** Comparison between ERTNet and previous work on DEAP (Subject-dependent).

Authors	Features	Classifier	Valence accuracy	Arousal accuracy
Koelstra et al. (2012)	Multimodal features	GNB	57.60%	62.00%
Zhong et al. (2022)	Multiscale sample entropy	CNN with HMM	**83.09%**	79.77%
Topic and Russo (2021)	Fusion features	SVM	76.61%	77.72%
Chao et al. (2019)	Multiband feature matrix	CapsNet	66.73%	68.28%
Samal and Hashmi (2023)	Features based MEEMD	SVM	74.3%	78.0%
Agrawal et al. (2023)	EEG signals	2D-CNN	66.99%	70.10%
Ding et al. (2022)	EEG signals	TSception	59.14%	61.57%
Ours	EEG signals	ERTNet	73.31%	**80.99%**

Bold values indicate the highest accuracy.

**Table 3:** Comparison between ERTNet and previous work on DEAP (Subject-Independent).

Authors	Features	Classifier	Valence accuracy	Arousal accuracy
Liang et al. (2019)	Muti-domain feature	UL	56.25%	62.34%
Appriou et al. (2020)	EEG signals	FBFgMDM	61.01%	60.30%
		FBTSC	61.09%	60.60%
Ding et al. (2022)	EEG signals	TSception	**62.27%**	63.75%
Ours	EEG signals	ERTNet	59.60%	**63.90%**

Bold values indicate the highest accuracy.

Taking the DEAP dataset-trained model as an example, we can visualize ERTNet’s feature extraction parameters as a signal and brain topology map to observe the model’s attention to various frequency bands and brain regions of the EEG signals. This also allows us to interpret the model’s internal learning. The conclusion is further supported by the results of the ablation experiments. In studies that aim to improve model interpretability, this often requires that the individual frequency bands of the EEG signal be extracted separately or that the EEG signal be time-frequency transformed before feeding into the model (Maheshwari et al., 2021). In clinical settings, achieving high accuracy and interpretability is of utmost importance (Ribeiro et al., 2016; Jafari et al., 2023). Black-box CNN models make it difficult to test decisions and fail to explain if their intrinsic representations correspond with clinical features, leading to doctors’ skepticism and hindering translational applications. Therefore, interpretability becomes a crucial factor, making ERTNet a more reliable option for clinical systems.

However, there is still some limitations in this study. Firstly, the utilization of two publicly accessible datasets with diminutive sample sizes, confined to controlled laboratory settings, potentially impedes the model’s generalizability. Future endeavors should encompass validation of the model’s efficacy on extensive medical EEG data and the acquisition of empirical data in multifaceted clinical environments for comprehensive assessment. Secondly, the exploration of a constrained range of parameter configurations in this study indicates potential for further refinement of the model’s architecture. The precision and interpretability of ERTNet could be augmented through the incorporation of ensemble learning techniques (Iyer et al., 2023) or the transmutation of domain-specific knowledge into feature (Bustios and Garcia Rosa, 2023). Furthermore, despite surpassing black-box models in interpretability, the explication process of ERTNet is not entirely lucid, necessitating future efforts to fortify its interpretability. Lastly, given that the dataset predominantly comprises data from a youthful and healthy demographic, it is imperative to ascertain the model’s robustness across diverse populations. In summation, while this study presents an interpretable and efficacious model for EEG emotion recognition tasks, the generalizability of the outcomes warrants additional validation, given the data, model, and interpretability constraints. This underscores the lifelong learning for ERTNet to ensure the model’s efficiency and reliability in complex clinical settings.

## 5 Conclusion

In this study, we developed an end-to-end transformer-based framework for EEG emotion recognition. This framework provides a local-to-global receptive field for the model by incorporating the transformer module with temporal–spatial convolution, significantly enhancing the accuracy of recognizing emotional information in the dimensional and discrete model, achieved through targeted tuning of the length of convolutional kernels. It shows that our model could provide better performance and interpretability in EEG emotion recognition.

However, our work still has limitations and we did not perform the same test on more datasets, especially private datasets. In future work, we hope to include more datasets for testing. Our related code will also be open-sourced on GitHub, and researchers are encouraged to test it on other datasets and publish their results.

## Data availability statement

The datasets presented in this study can be found in online repositories. The names of the repository/repositories and accession number(s) can be found at: http://www.eecs.qmul.ac.uk/mmv/datasets/deap/download.html, https://bcmi.sjtu.edu.cn/~seed/seed-v.html, and the code for this study can be found at: https://github.com/ruix6/ERTNet.

## Author contributions

RL: Data curation, Formal analysis, Investigation, Methodology, Software, Visualization, Writing – original draft, Writing – review & editing. YC: Conceptualization, Formal analysis, Resources, Writing – review & editing, Supervision. XM: Software, Validation, Writing – review & editing. SC: Conceptualization, Funding acquisition, Project administration, Resources, Supervision, Writing – review & editing. XS: Writing – review & editing. LS: Writing – review & editing. SL: Supervision, Writing – review & editing.
