# Supplementary material for: ERTNet: an interpretable transformer-based framework for EEG emotion recognition
Source: Front Neurosci. 2024 Jan 17;18:1320645. doi: 10.3389/fnins.2024.1320645 (PMC10827927; doi:10.3389/fnins.2024.1320645)
Supplement: Supplementary file 1 [file Table_1.pdf]

1 Appendix

The structures of EEGNet, DeepConvNet, ShallowConvNet, and CNN-BiLSTM are described in detail in Tables 4, 5, 6, and 7. EEGNet is a specialized, compact convolutional neural network used for processing EEG signals, while ShallowConvNet is intended for oscillatory signals. ShallowConvNet is optimized for handling oscillatory signals, while DeepConvNet is a generalized CNN architecture. However, the convolutional neural network's perceptual field is inadequate for capturing contextual information in EEG. CNN-BiLSTM amalgamates CNN and LSTM to augment the perceptual field of CNN. However, the model is vulnerable to overfitting.

Table 4. EEGNet structure, where F_1 = number of temporal kernels, T = length of temporal kernels, D = number of spatial kernels, C = number of channels, F_2 = number of pointwise kernels, N = number of classes, and S = length of sample.

Layer	Filters	Size
Input		$(C, S, 1)$
Conv2D<br>BatchNorm	F_1	$(1, T)$
DepthwiseConv2D<br>BatchNorm<br>Activation (ELU)	$D \times F_1$	$(C, 1)$
AveragePool2D		(1, 4)
Dropout		
SeparableConv2D<br>BatchNorm<br>Activation (ELU)	F_2	(1, 16)
AveragePool2D		(1, 8)
Dropout		

Flatten		
Dense	$N \times (F_2 \times S//32)$	

Table 5. DeepConvNet structure, where C = number of channels, S = length of sample, and N = number of classes.

Layer	Filters	Size
Input		$(C,\ S, 1)$
Conv2D	25	(1, 5)
Conv2D<br>BatchNorm<br>Activation (ELU)<br>MaxPool2D	25	$(C, 1)$<br><br><br>(1, 2)
Dropout		
Conv2D<br>BatchNorm<br>Activation (ELU)<br>MaxPool2D	100	(1, 5)<br><br><br>(1, 2)
Dropout		
Conv2D<br>BatchNorm<br>Activation (ELU)<br>MaxPool2D	50	(1, 5)<br><br><br>(1, 2)
Dropout		

Conv2D<br>BatchNorm<br>Activation (ELU)<br>MaxPool2D	100	(1, 5)<br><br><br>(1, 2)
Dropout		
Conv2D<br>BatchNorm<br>Activation (ELU)<br>MaxPool2D	200	(1, 5)<br><br><br>(1, 2)
Dropout		
Flatten		
Dense	N	

Table 6. ShallowConvNet structure, where C = number of channels, S = length of sample, and N = number of classes. The 'square' and 'log' activation functions are given as $f(x) = x^2$ and $f(x) = log(x)$, respectively.

Layer	Filters	Size
Input		$(C,\ S, 1)$
Conv2D	40	(1, 13)
Conv2D<br>BatchNorm<br>Activation (square)	40	$(C, 1)$
AveragePool2D		(1, 35), stride (1, 7)
Activation (log)		

Flatten		
Dropout		
Dense	N	

Table 7. CNN-BiLSTM structure, where F_1 = number of temporal kernels, T = length of temporal kernels, D = number of spatial kernels, C = number of channels, F_2 = number of pointwise kernels, N = number of classes, and S = length of sample.

Layer	Filters	Size
Input		$(C, S, 1)$
Conv2D<br>BatchNorm	F_1	$(1, T)$
DepthwiseConv2D<br>BatchNorm<br>Activation (ELU)	$D \times F_1$	$(C, 1)$
AveragePool2D		(1, 4)
Dropout		
SeparableConv2D<br>BatchNorm<br>Activation (ELU)	F_2	(1, 16)
TimeDistributed (Flatten)		
Bidirectional (LSTM)	64	
Flatten		
Dropout		

Dense	*N*	
